# Identification of Lead Compounds against Scm (fms10) in *Enterococcus faecium* Using Computer Aided Drug Designing

**DOI:** 10.3390/life11020077

**Published:** 2021-01-21

**Authors:** Muhammad Asif Rasheed, Muhammad Nasir Iqbal, Salina Saddick, Iqra Ali, Falak Sher Khan, Sumaira Kanwal, Dawood Ahmed, Muhammad Ibrahim, Umara Afzal, Muhammad Awais

**Affiliations:** 1 Department of Biosciences, Sahiwal Campus, COMSATS University Islamabad, Sahiwal 57000, Pakistan; asif.rasheed@cuisahiwal.edu.pk (M.A.R.); nasiriqbal786@hotmail.com (M.N.I.); iqraali857@gmail.com (I.A.); sumaira.kanwal@cuisahiwal.edu.pk (S.K.); ibrahim@cuisahiwal.edu.pk (M.I.); 2 Department of Biological Sciences, Faculty of Science, King Abdulaziz University, P.O. Box 80203, Jeddah 21589, Saudi Arabia; sghoth@kau.edu.sa; 3 Department of Biotechnology, University of Sialkot, Sialkot 51040, Pakistan; Falak.sher@uskt.edu.pk; 4 Department of Medical Lab Technology, University of Haripur, Haripur 22620, Pakistan; dawood@uoh.edu.pk; 5 Department of Chemistry, Rawalpindi Women University, Satellite Town, Rawalpindi 43600, Pakistan; uachem@f.rwu.edu.pk; 6 University Institute of Biochemistry and Biotechnology (UIBB), PMAS-Arid Agriculture University Rawalpindi, Rawalpindi 43600, Pakistan

**Keywords:** *Enterococcus faecium* DO, drug designing, virtual screening, bioinformatics

## Abstract

(1) Background: *Enterococcus faecium* DO is an environmental microbe, which is a mesophilic, facultative, Gram-positive, and multiple habitat microorganism. *Enterococcus faecium* DO is responsible for many diseases in human. The fight against infectious diseases is confronted by the development of multiple drug resistance in *E. faecium*. The focus of this research work is to identify a novel compound against this pathogen by using bioinformatics tools and technology. (2) Methods: We screened the proteome (accession No. PRJNA55353) information from the genome database of the National Centre for Biotechnology Information (NCBI) and suggested a potential drug target. I-TASSER was used to predict the three-dimensional structure of the protein, and the structure was optimized and minimized by different tools. PubChem and ChEBI were used to retrieve the inhibitors. Pharmacophore modeling and virtual screening were performed to identify novel compounds. Binding interactions of compounds with target protein were checked using LigPlot. pkCSM, SwissADME, and ProTox-II were used for adsorption, distribution, metabolism, excretion, and toxicity (ADMET) properties. (3) Results: Novel selected compounds have improved absorption and have better ADMET properties. Based on our results, the chemically identified inhibitor ZINC48942 targeted the receptor that can inhibit the activity of infection in *E. faecium*. This research work will be beneficial for the scientific community and could aid in the design of a new drug against *E. faecium* infections. (4) Conclusions: It was observed that novel compounds are potential inhibitors with more efficacy and fewer side effects. This research work will help researchers in testing and identification of these chemicals useful against *E. faecium.*

## 1. Introduction

In the early 1900s, *Enterococcus faecalis* and *faecium* were identified and isolated. In human beings, these are the most abundant species comprising up to one percent of microbiota in the intestine [1]. To treat diseases (neonatal meningitis, urinary tract infections, surgical wound infections, nosocomial bacteremia, and catheter-related) caused by E. *faecium,* billions of dollars are spent, and this pathogen kills roughly two million people every year. Some strains may additionally cause endocarditis, intra-abdominal, and pelvic infections.

Organisms are transmitted via direct contact. The use of broad-spectrum antibiotics and devices are the major aspects contributing to the emergence of *E. faecium* as a significant pathogen. The pathogenic strains can cause sickness in even the healthiest host [1,2].

In nature, *E. faecium* is basically part of the normal intestinal flora, but it also occurs in food. The intestine of animals, including human beings, are perfect places for gene transfer. *E. faecium* show the capability to take up and transfer antibiotic resistance genes, both vertically and horizontally [3]. The urinary tract infection is caused by Enterococcus, a Gram-positive cocci. Males (33.96%) are less prone to enterococcal infection as compared to females (66.04%), and the most affected age group by this pathogen is 21–30 years [4]. The most common enterococcal species present various virulence factors, such as collagen binding protein, cytolysin, enterococcal surface protein, gelatinase, and aggregation substance [5].

The resistance of bacteria to antibiotics is a common phenomenon, but for human beings, it is becoming a serious threat. Because of antibiotic-resistant microorganisms, at least 23,000 people died every year in the United States of America. By 2050, antibiotic-resistant will cause around 300 million premature deaths and the global economy may suffer a loss of up to $100 trillion [6]. Thirty-three percent of enterococcal infections are drug-resistant, and there are 20,000 cases of antibiotic resistance yearly. Many *Enterococcus faecium* strains show resistance to penicillin, ampicillin, daptomycin, gentamicin, tetracycline, and vancomycin. The treatment of infections caused by enterococcal becomes very difficult due to antibiotic resistance [1].

The antibiotic resistance genes of *Enterococci* in human beings may be spread through the consumption of vegetables and animals. Moreover, *Enterococci* could spread antibiotic resistance genes to their own or other species, with the possibility of causing endogenous infections. Consequently, transferring microbes containing antibiotic resistance genes between humans and animals is mostly done by the food chain [7].

During growth, vertical transmission of chromosomal mutations is the reason for the spread of antibacterial resistance. Genetic recombination of DNA and the food chain is the main cause of horizontal genetic exchange of resistance genes. The occurrence of antimicrobial resistance mostly depends on host species and country of origin. When a resistance mechanism encoded by a single gene carries resistance to multiple antibacterial compounds, then cross-resistance to several antibacterial may additionally arise [8]. Already present antibiotics show resistance, so there is a need to introduce new antimicrobial compounds to overcome diseases caused by *Enterococcus faecium*.

Recently it has been proved that *E. faecium* show high-level resistance to most beneficial anti-enterococcal antibiotics, ampicillin, vancomycin, and aminoglycosides. Because of the low success rate, some new antibiotics, such as linezolid, daptomycin, and tigecycline, have limited clinical use. However, these antibiotics show a good success rate in laboratory experiments against enterococcal isolates. So, treatment of infections caused by enterococcal multidrug-resistance is based on unprejudiced observations and deductions from wet-lab experiments. We need urgent strategies to develop more efficient therapies to deal with intense multidrug-resistant *E. faecium* infections [9].

Infections, spread through different microbes, are the main threat to human existence. To cope with this enormously increasing danger of multidrug resistance, we must develop new strategies to find novel potential drug candidates. Traditional techniques of drug discovery are monotonous, extremely time-consuming, and uneconomical. These techniques are also a threat to the principles of green chemistry. Subsequently, the tools and techniques of bioinformatics have gained research attention to lessen the time and cost by computer-aided drug design [10].

The capacity of *E. faecium* to cause persistent disease is an essential property of the organism. Hospital-associated infections are amplified by the spread of drug-resistant *Enterococci* in the hospital setting. [11]. In this context, the aim of our study is to propose new antimicrobial compounds against *E. faecium,* to inhibit the pathogen’s functionality by targeting a bacterial protein and to predict lead compounds against scm (fms10) of *E. faecium.*

## 2. Materials and Methods

**Figure 1:**
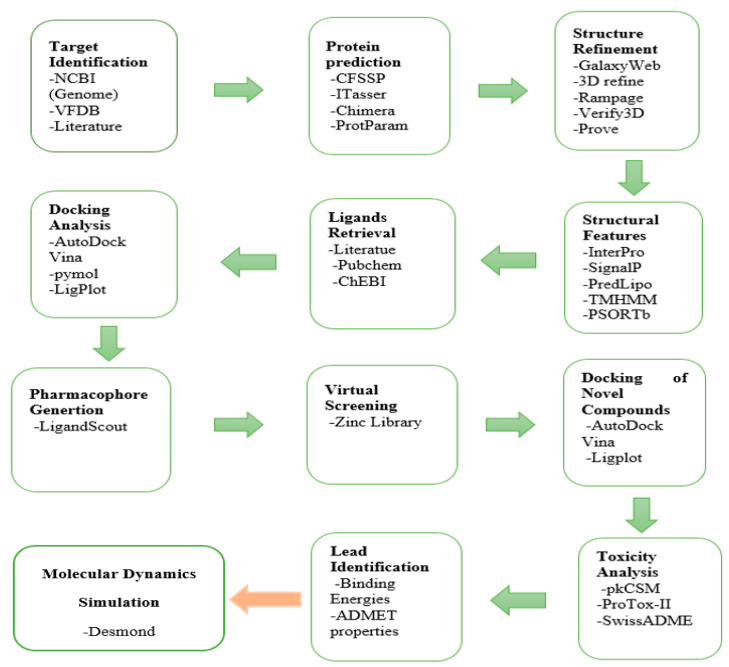
Flowchart of Adopted computer-aided drug design (CADD) Methodology.

The flowchart of the adopted computer-aided drug design (CADD) is given in Figure 1. The details of the methodology are as follows.

### 2.1. Target Identification

*E. faecium* contains one chromosome and three plasmids, 3209 genes, which translate into 3114 proteins, and the Gas Chromatography (GC) content is 37.8% [12]. Four out of 22 surface proteins of *E. faecium* show a virulence factor. Against infectious diseases caused by *Enterococcus faecium* potential drug targets, recombinant Scm65 (A- and B-domains) and Scm36 (A-domain) are identified, as revealed by functional annotation using various computational tools and techniques [13], were used. For virulence factor analysis, the Virulence Factor Database (VFDB) was used [14].

### 2.2. Protein Selection and Structural Refinement

To retrieve protein sequences in FASTA format, UniProt (Universal Protein Resource) database (http:/www.uniprot.org/) was used [15]. Expasy ProtParam and CFSSP (http://www.biogem.org/tool/chou-fasman/) was used to calculate physiochemical properties of protein and prediction of secondary structure, respectively [16]. This also helps in the prediction of protein functFns [17]. The target protein structure was not present in PDB, and the sequence of the target protein did not fulfill the requirements of homology modeling, therefore, we moved toward threading based modeling, and for this purpose, we used the online server I TASSER [18]. We used UCSF Chimera for protein visualization [19].

For structure refinement, we used two online servers, named GalaxyWeb and 3Drefine [20,21]. For generating the Ramachandran plot, Rampage was used, The plot also provides information about the residues lying in favored, allowed, or outlier regions [22]. To evaluate theoretical protein models, QMEAN Z-score was used [23]. PROVE (PROtein Volume Evaluation) was used for structure validation procedures [24]. VERIFY3D was used to verify the final model of the predicted protein [25].

### 2.3. Protein Properties

InterPro and PRED-LIPO, a Hidden Markov Model, was used to classify the protein domain and lipoprotein signals, respectively [26,27]. Trans-membrane helixes were predicted by using the TMHMM server v.2.0 [28]. PSORTb was used for the prediction of prokaryotic localization sites [29]. Binding and active sites were predicted using COFACTOR and CASTp [30,31].

### 2.4. Selection and Retrieval of Ligands

Overall, 211 Chemical compounds from PubChem, ChEBI, and Literature Survey were considered as ligands considering their biological activities. The 2D chemical structure was retrieved and converted into 3D by using Discovery Studio.

### 2.5. Docking Analysis

Docking results were obtained according to their binding affinities by using AutoDock Vina. PyMOL and Discovery Studio were used for the analysis of the protein–ligand complex to understand the interactions between receptor and inhibitor along with the binding sites of target Protein.

### 2.6. Pharmacophore Generation

The selected compounds with a wide range of structural diversity and activity were aligned. A pharmacophore model was generated to merge all the features of selected compounds. The pharmacophore of the top 10 inhibitors against targeted Scm (Fms10) was generated using LigandScout. Virtual screening was performed against the ZINC database to find the inhibitors which can inhibit adherence activity [32,33].

### 2.7. Docking of Novel Compounds

All identified hits of pharmacophore-based virtual screening were sorted according to their pharmacophore-fit score, and 100 compounds were selected and filter on the basis of two rules, named rule of five and the Veber rule. Then the top 15 compounds were selected. These compounds were docked with the receptor and evaluated for binding energies and protein–ligand interactions by using AutoDock Vina. Pymol was used for making complex files of receptor and ligand, and for finding interactions, LigPlot was used, respectively.

### 2.8. Toxicity Analysis

After a thorough analysis of docking results, drug likeness and toxicity characteristics were identified through pkCSM [34], ProTox-II [35], and SwissADME [36], which are reported as useful tools in calculating important drug-like descriptors, such as adsorption, distribution, metabolism, excretion, and toxicity (ADMET), as well as use for predicting lead likeness with respect to mutagenicity and carcinogenicity.

### 2.9. Lead Identification

The most active inhibitors were identified based on docking score, ligand–protein interactions, and toxicity analysis studies including Molecular Weight (MW), Hydrogen Bond Donner (HBD), Hydrogen Bond Acceptor (HBA), partial coefficient logP, Polar Surface Area (PSA), rotatable bonds, rings, Blood–Brain Barrier and Ames Toxicity etc. The compounds showing the least binding affinity, high lead likenesses, and best interactions were selected as potential inhibitors of Scm (Fms10).

### 2.10. Molecular Dynamics Simulation

Molecular dynamics simulations were performed for 50 nanoseconds using Desmond, a Package of Schrödinger LLC [37]. The initial stage of protein and ligand complexes for molecular dynamics simulation were obtained from docking studies. Molecular Docking Studies provide a prediction of ligand binding status in static conditions. Simulations were carried out to predict the ligand binding status in the physiological environment. The protein–ligand complexes were preprocessed using Protein Preparation Wizard or Maestro, which also included optimization and minimization of complexes. All systems were prepared by the System Builder tool. Solvent Model with an orthorhombic box was selected as TIP3P (Transferable Intermolecular Interaction Potential 3 Points). The OPLS_2005 force field was used in the simulation [38]. The models were made neutral by adding counter ions where needed. To mimic the physiological conditions, 0.15 M salt (NaCl) was added. The NPT ensemble (Isothermal-Isobaric: moles (N), pressure (P), and temperature (T) are conserved) with 300 K temperature and 1 atm pressure was select for complete simulation. The models were relaxed before the simulation. The trajectories were saved after every 50 ps for analysis, and the stability of simulations was evaluated by calculating the root mean square deviation (RMSD) of the protein and ligand over time.

## 3. Results and Discussion

**Figure 2:**
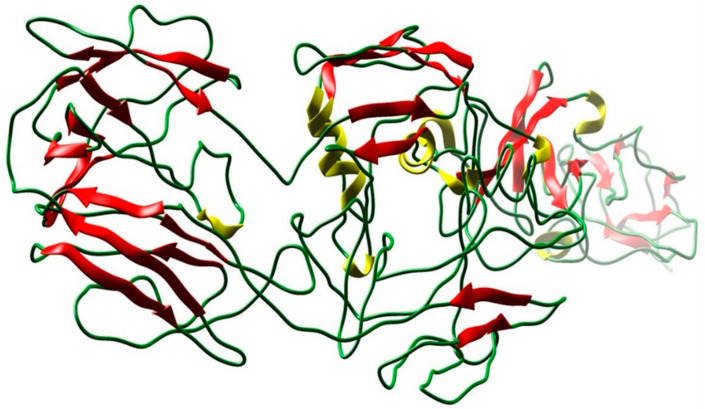
3D structure of the target protein predicted by I-TASSER using homology modeling (UniProt ID: 13U5K9).

The amino acid sequence of target proteins of *Enterococcus faecium* was retrieved from the UniProt database (I3U5K9). The structure of the protein was generated by I-TASSER, shown in Figure 2.

**Figure 3:**
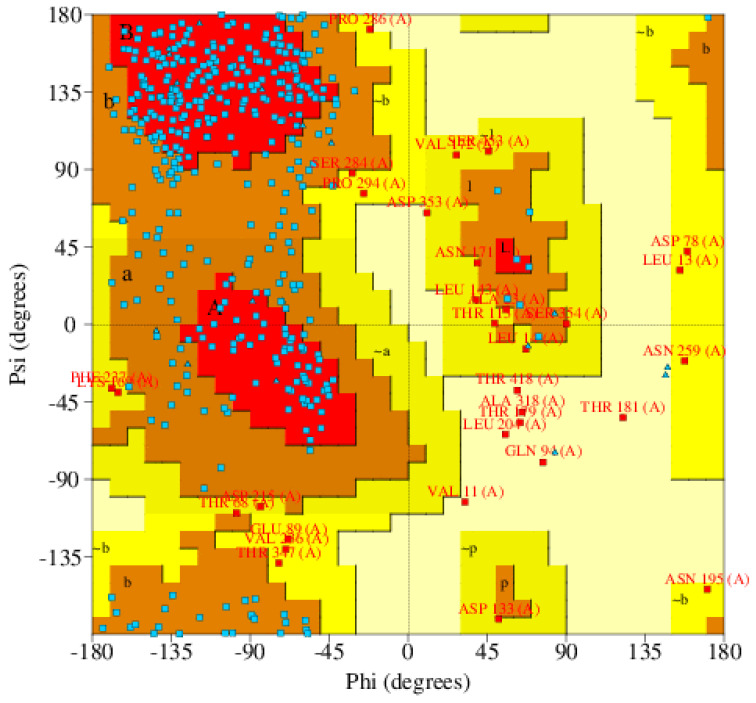
Ramachandran Plot, showing different regions of protein structure.

**Figure 4:**
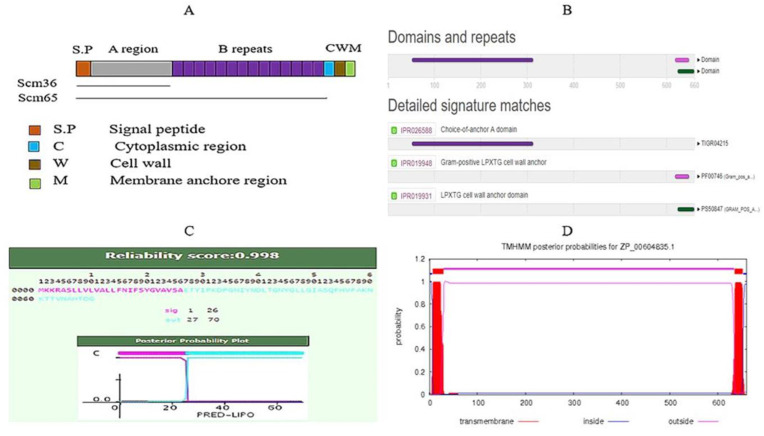
Structural features. (**A**) Subcellular localization. (**B**) Domains and repeats by InterPro. (**C**) Lipoprotein Signal Peptide. (**D**) Prediction of Transmembrane Helices.

The overall quality of the structure was 90.1% using Rampage, as mentioned in Table 1 and Figure 3. The predicted structure contains 24 helix, 36 sheets, and 40 coils. Scm (Fms10) of *E. faecium* DO has total 14 B-repeats from which 13 beta-repeats of 19 residues in length and 1 partial repeat of 10 residues also contain a signal peptide, and if we see subcellular localization in Figure 4A, then a major part of protein lies in cell wall region. Domains and functional sites and lipoprotein signal peptides of the target protein are shown in Figure 4B,C, respectively.

The Hidden Markov Model method was used for the prediction of lipoprotein signal peptides of Gram-positive bacteria. Transmembrane helices were predicted by using TMHMM server v 2.0. According to the Exp number of amino acids in trans-membrane helices (TMHs), the expected number of amino acids in transmembrane helices can be determined. If this number is larger than 18, it is very likely to be a transmembrane protein or have a signal peptide. Total prob of N-in: The total probability that the N-term is on the cytoplasmic side of the membrane. The bacterial protein value of the expected amino acids was more than 18, and almost all graphs had the same protruded area into and outside the cell and the transmembrane area, see Figure 4D.

There were 211 inhibitors retrieved against Collagen-binding MSCRAMM Scm (Fms10) from PubChem, ChEBI, and Literature. All these were selected based on their inhibitory effect on Collagen-binding MSCRAMM Scm (Fms10) involved in multi-drug resistance. Among 211 compounds, 161 failed during Lipinski and Veber filtering (such as Molecular Weight > 500, logP > 5, H-Bond Donors > 5, H-Bond acceptors > 10, PSA < 140, RB < 10).

The selected inhibitors were docked with Collagen-binding MSCRAMM Scm (Fms10), and 10 ligands with best binding affinities were chosen, Table 2. These inhibitors were analyzed through LigPlot to determine the amino acids involved in protein–ligand binding interactions.

**Figure 5:**
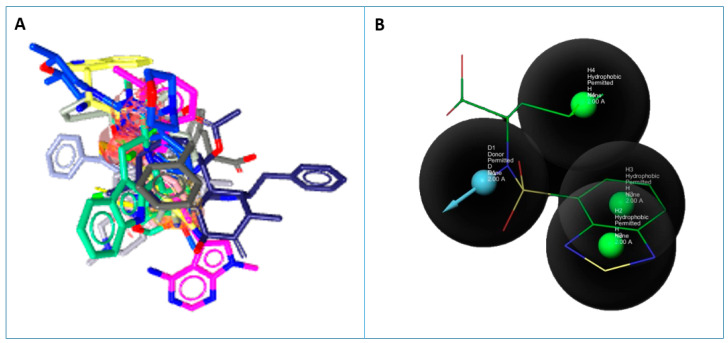
Ligand based pharmacophore. (**A**) Aligned ligands. (**B**) Selected ligand fit on pharmacophore hypothesis.

Based on binding energies, the top 10 selected inhibitors were chosen to generate a pharmacophore model. This merged pharmacophore model with matching features, such as Hydrogen Bond Donors, Hydrogen Bond Acceptors, and Aromatic Rings, is shown in Figure 5.

After pharmacophore modeling, virtual screening using the ZINC library was performed via LigandScout, to identify the compounds with features like those of the pharmacophore model. Twenty-one thousand, three hundred and thirty-one hits (Hitrate: 70%) were identified out of 30,737 compounds. Based on the Pharmacophore-fit score, the top 100 compounds were selected, and again the rule of five and Veber rule was applied. After applying both rules, 15 novel compounds were selected for molecular docking with the target protein. Molecular docking of Scm (Fms10) with selected novel compounds was conducted using AutoDock Vina (Table 3).

**Figure 6:**
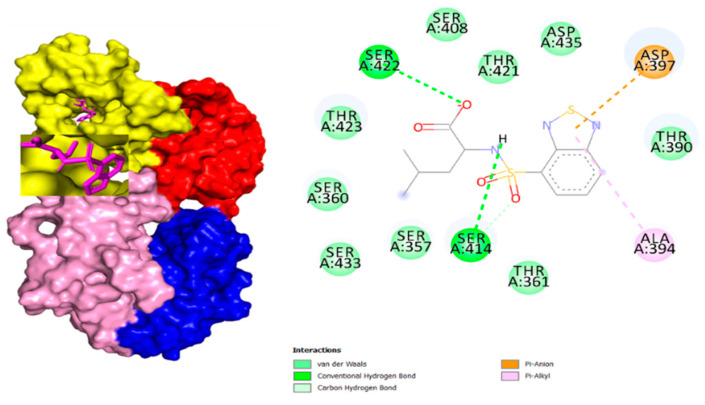
Binding Interactions of ZINC48942 with Scm.

After docking through autodock vina, the ADMET properties of novel compounds were determined, and one compound (ZINC48942) was identified as the most active from all molecules after toxicity analysis. Properties and interactions of the best one are shown in Table 4 and Figure 6.

**Figure 7:**
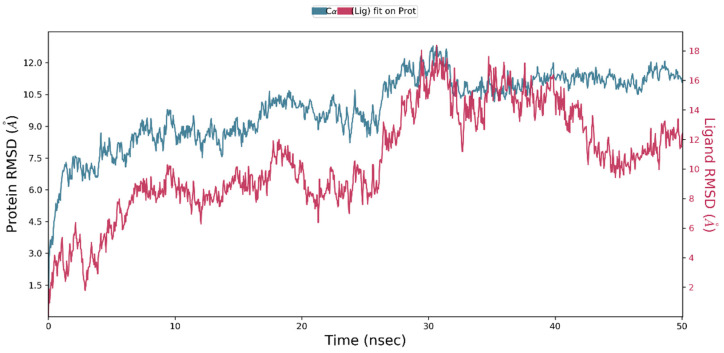
Root mean square deviation (RMSD) of the backbone atoms of protein and the ligand with time. The left *Y*-axis shows the variation of protein RMSD through time. The right *Y*-axis shows the variation of ligand RMSD through time.

The Desmond simulation trajectories were analyzed. Root mean square deviation (RMSD), root mean square fluctuation (RMSF), and protein–ligand contacts were calculated from MD trajectory analysis. Figure 7 shows the evolution of RMSD values in the course of time for the backbone atoms of the ligand bound protein. The RMSD plot of the complex indicates that the complex reaches stability at 10 ns. From then, an average RMSD value of 2.2 Å persists up to 50 ns. After that, changes in RMSD values remain within 2.2 Å during the simulation period, which is quite acceptable for small, predicted proteins. Ligand fit to protein RMSD values fluctuates within 1.0 Angstrom after being stable. These indicate that the ligand remains stably bound to the binding site of the receptor during the simulation period.

**Figure 8:**
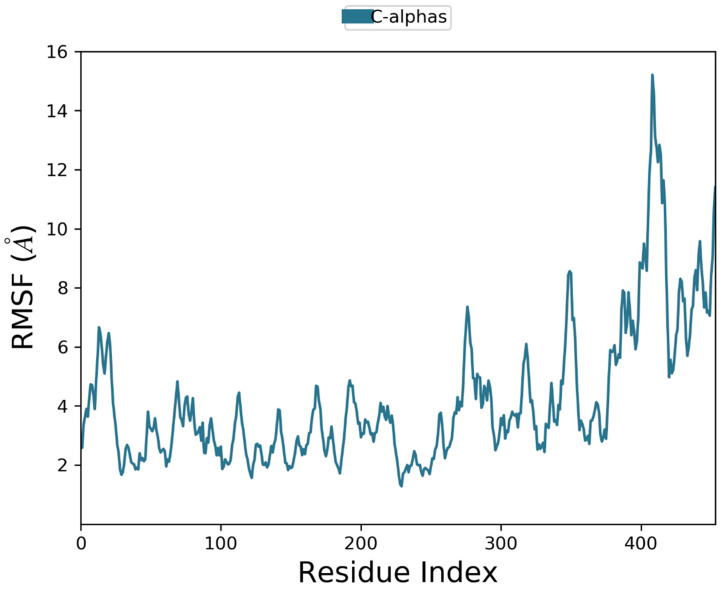
Residue wise Root Mean Square Fluctuation (RMSF) of protein.

**Figure 9:**
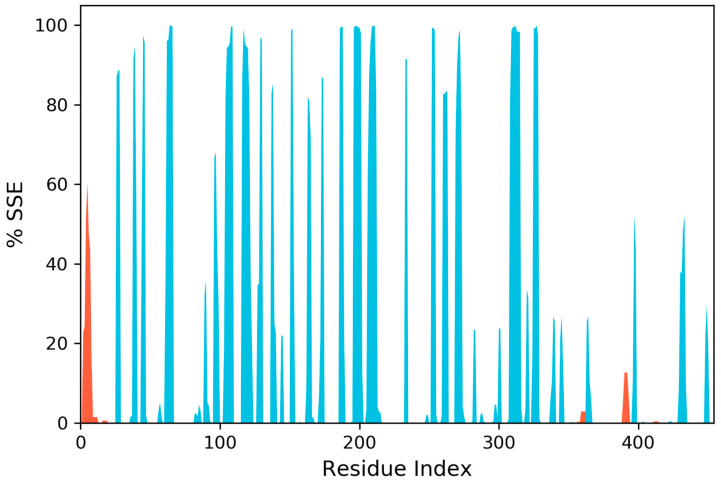
Protein Secondary Structure element distribution by residue index throughout the protein structure. Red columns indicate alpha helices, and blue columns indicate beta-strands.

Figure 8 shows the residue wise RMSF value of the protein bound to the ligand. The residues showing higher peaks correspond to loop regions, as identified from MD trajectories (Figure 9), or N and C-terminal zones. Low RMSF values of binding site residues indicate the stability of ligand binding to the protein.

**Figure 10:**
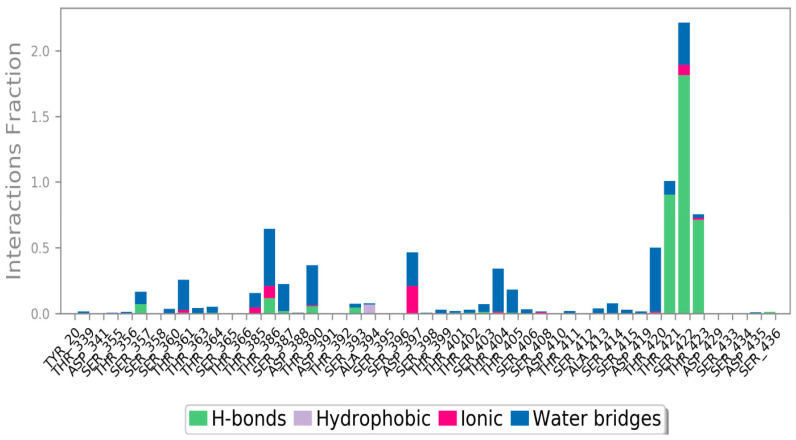
Protein-ligand contact histogram.

Most of the important interactions of ligand–proteins determined with MD are hydrogen bonds and hydrophobic interactions, as depicted in Figure 10. THR_421, SER_22, and THR_423 are the most important ones in terms of H-bonds. The stacked bar charts were normalized over the course of the trajectory: for example, a value of 1.0 suggests that for 100% of the simulation time, the specific interaction was maintained. Values over 1.0 are possible as some protein residue may make multiple contacts of the same subtype with the ligand.

## 4. Conclusions

Several multidisciplinary methods have gained research attention to lessen the time and cost during the drug development process. The motivation of this research work was to find target proteins and then select inhibitors for infectious *Enterococcus faecium* strains. From the ZINC database, we selected chemical compounds that inhibit the effect of the Scm (Fms10) protein. Pharmacophore modeling with virtual screening and docking analysis helped to separate the compounds having the least binding energy with the target protein. The chemically identified inhibitor ZINC48942 targeted the receptor that can inhibit the activity of adherence and spreading of infection in *E. faecium.* We concluded that this drug could be used as a lead compound to develop a drug that can selectively act against *E. faecium* infections without interfering with the activities of the human proteasome. These findings will be beneficial for the scientific community and could aid in the design of a new drug against *E. faecium* infections.

## Figures and Tables

**Table 1:** Scores of GalaxyWEB and 3Drefine and Refinement Databases and PSORTb.

UniProt Ids	I3U5K9
**GalaxyWEB and 3Drefine**	
GDT-HA	0.9992
RMSD	0.448
MolProbity	3.054
3Drefine Score	42,972.1
Rama favored	89.7
**Refinement Databases Score**	
Qmean	−6.87
Prove	6.9
Rampage (favored region)	90.1
Verify 3D	60.70
**Subcellular Localization**	
Cell Wall	9.98
Cytoplasmic Membrane	0.01
Extracellular	0.001
Cytoplasmic	0.0001
Final Prediction	Cell Wall

**Table 2:** Docking results and 2D structures of top 10 inhibitors.

Ligand ID’s	Binding Energies	No. of H-Bonds	2D Structures
71940	−10	1	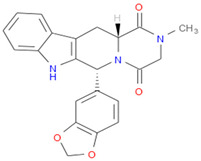
85572	−9.4	2	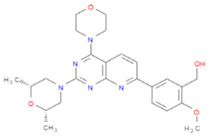
131686	−9.3	1	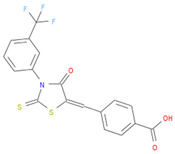
139047	−9.1	2	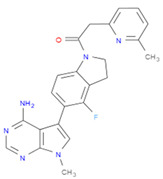
140296	−9.1	1	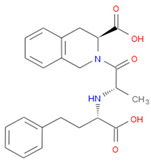
63598	−8.6	1	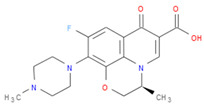
104872	−8.2	1	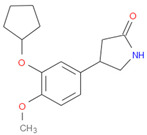
137693	−8.2	1	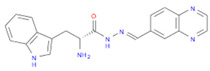
43541	−8.1	1	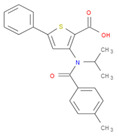
529996	−8.1	1	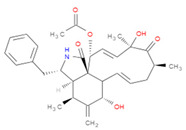

**Table 3:** Features of Novel Compounds after Virtual Screening.

Zinc IDs	Pharma-Cophore Score	Binding Affinity	HBA	HBD	Rings	RBs	M.W	logP	TPSA
zinc_5347917	43.62	−8.8	5	2	4	8	406.39	1.43	124.88
zinc_5223045	43.96	−8.2	8	2	5	8	452.49	2.08	93.06
zinc_12603547	43.66	−7.8	6	5	3	9	417.44	0.86	119.92
zinc_49590510	43.78	−7.2	5	0	4	8	433.53	1.21	82.03
zinc_77264335	43.71	−7.2	5	3	3	7	493.71	4.19	102.76
zinc_1440812	43.62	−7.1	5	2	3	9	358.35	0.93	124.88
zinc_72321048	43.62	−7.1	5	3	3	6	362.4	1.83	104.73
zinc_57855	43.67	−7	4	1	3	4	270.28	2.21	55.76
zinc_12603524	43.79	−7	7	4	3	9	420.41	0.37	107.89
zinc_35425432	43.62	−6.7	7	1	4	9	382.44	0.95	86.4
zinc_57662	43.89	−6.6	3	0	3	5	282.29	3.53	48.67
zinc_48942	43.78	−6.4	6	1	2	8	328.39	0.14	112.08
zinc_3897410	43.97	−6.1	6	4	2	7	268.24	−3.69	122.08
zinc_12603765	43.77	−5.6	6	5	2	8	350.35	−0.97	107.89
zinc_3839734	44.2	−4.7	5	3	2	8	399.49	1.07	118.8

**Table 4:** Properties of Lead Compound.

Compounds ID	Zinc_48942
Pharmacophore Score	43.67
Molecular Weight	270.28
H-bond Donor	4
H-bond Acceptor	1
Rotatable bonds	4
Rings	3
Toxicity	Non-Toxic
Carcinogenetic	Non-Carcinogen
Binding Energy	−7
No. of H-Bonds	2
Interacting Residues	NDI-His67:O15; N-Ala66:O15
Distance	2.99 2.89

## Data Availability

Not applicable.
